# Role of calcium supplementation during pregnancy in reducing risk of developing gestational hypertensive disorders: a meta-analysis of studies from developing countries

**DOI:** 10.1186/1471-2458-11-S3-S18

**Published:** 2011-04-13

**Authors:** Aamer Imdad, Afshan Jabeen, Zulfiqar A Bhutta

**Affiliations:** 1 Division of Women & Child Health, The Aga Khan University, Karachi, Pakistan

## Abstract

**Background:**

Hypertension in pregnancy stand alone or with proteinuria is one of the leading causes of maternal mortality and morbidity in the world. Epidemiological and clinical studies have shown that an inverse relationship exists between calcium intake and development of hypertension in pregnancy though the effect varies based on baseline calcium intake and pre-existing risk factors. The purpose of this review was to evaluate preventive effect of calcium supplementation during pregnancy on gestational hypertensive disorders and related maternal and neonatal mortality in developing countries.

**Methods:**

A literature search was carried out on PubMed, Cochrane Library and WHO regional databases. Data were extracted into a standardized excel sheet. Identified studies were graded based on strengths and limitations of studies. All the included studies were from developing countries. Meta-analyses were generated where data were available from more than one study for an outcome. Primary outcomes were maternal mortality, eclampsia, pre-eclampsia, and severe preeclampsia. Neonatal outcomes like neonatal mortality, preterm birth, small for gestational age and low birth weight were also evaluated. We followed standardized guidelines of Child Health Epidemiology Reference Group (CHERG) to generate estimates of effectiveness of calcium supplementation during pregnancy in reducing maternal and neonatal mortality in developing countries, for inclusion in the Lives Saved Tool (LiST).

**Results:**

Data from 10 randomized controlled trials were included in this review. Pooled analysis showed that calcium supplementation during pregnancy was associated with a significant reduction of 45% in risk of gestational hypertension [Relative risk (RR) 0.55; 95 % confidence interval (CI) 0.36-0.85] and 59% in the risk of pre-eclampsia [RR 0.41; 95 % CI 0.24-0.69] in developing countries. Calcium supplementation during pregnancy was also associated with a significant reduction in neonatal mortality [RR 0.70; 95 % CI 0.56-0.88] and risk of pre-term birth [RR 0.88, 95 % CI 0.78-0.99]. Recommendations for LiST for reduction in maternal mortality were based on risk reduction in gestational hypertensive related severe morbidity/mortality [RR 0.80; 95% CI 0.70-0.91] and that for neonatal mortality were based on risk reduction in all-cause neonatal mortality [RR 0.70; 95% CI 0.56-0.88].

**Conclusion:**

Calcium supplementation during pregnancy is associated with a reduction in risk of gestational hypertension, pre-eclampsia neonatal mortality and pre-term birth in developing countries.

## Background

Hypertension in pregnancy stand alone or with proteinuria is one of the leading causes of maternal mortality and morbidity in the world [1]. Hypertensive disorders are the second most common cause of maternal deaths worldwide [2] and account for more than 40,000 maternal deaths annually [3]. These disorders are also associated with adverse perinatal outcomes such as stillbirth, preterm and small for gestational age babies [4-6].

Epidemiological and clinical studies have shown that an inverse relationship exists between calcium intake and development of hypertension in pregnancy [4,7]. Many trials have been conducted to observe the protective effect of preventive calcium supplementation in pregnant women [8]. There is substantial data that supports that calcium supplementation in pregnancy is associated with reduction in gestational hypertensive disorder [9,10], although the impact varies according to the baseline calcium intake of the population and pre-existing risk factors [8,11].

A previous review by Hofmyer et al. has shown that calcium supplementation during pregnancy had a significant effect in reducing risk of gestational hypertension and pre-eclampsia [10]. This effect was more prominent in those studies where participants had low baseline calcium intake compared to that of adequate calcium intake [10]. Another review by Trumbo et al. had shown that beneficial effects of calcium supplementation cannot be generalized to USA population and suggested that beneficial effects could only be shown in populations whose baseline calcium intake is inadequate [11].

The objective of this review was to evaluate the effect of calcium supplementation during pregnancy in reducing maternal hypertensive disorders and related maternal and neonatal mortality and morbidity in developing countries. This paper is a part of series of papers for Lives Saved Tool (LiST) model. An intervention is currently included in the LiST if there is substantial evidence that it decrease maternal mortality, neonatal/child mortality and/or stillbirths [12]. This process is guided by qualitative assessment of available evidence according to Grading of Recommendations, Assessment, Development and Evaluation (GRADE) criteria [13] and quantitative inputs according to Child Health Epidemiology Reference Group (CHERG) guidelines [12]. For more details of the review methods, the adapted GRADE approach or the LiST model, see the methods and results section and other articles in this supplement.

## Methods

### Searching

To identify studies that evaluated the effect of calcium supplementation during pregnancy for prevention of gestational hypertensive disorders, a comprehensive search of PubMed, Cochrane Library and WHO regional databases was carried out using different terms for calcium and gestational hypertensive disorders. Following search strategy was used for PubMed; (“*Calcium” AND “pregnancy”*) *AND* (*“Hypertension” OR “pre-eclampsia” OR “blood pressure” OR “neonatal death” OR “preterm “OR “low birth weight”*). The search was limited to “randomized trial” and “humans”. Studies were considered for inclusion irrespective of language or status of publication. The date of last search was March 22, 2010. Additional studies were obtained through hand search of references from identified studies and previous reviews.

### Inclusion/exclusion criteria

All published randomized controlled trials (RCTs) in which pregnant women received calcium supplementation as compared to control (placebo or no intervention) were included. Observational studies were not considered for inclusion as we expected to have reasonable number of RCTs for inclusion in the review. Trials which included participants diagnosed with hypertension prior to pregnancy were excluded. Supplementation started with calcium before 32 weeks of pregnancy at the latest. As per objectives of LiST model, all the included studies were from developing countries [12]. The developing countries were defined as countries with Gross National Income per capita (GNI) below US$11,905, according to World Bank[14]. High blood pressure was taken as defined by authors mostly as diastolic blood pressure equal to or greater than 90 mmHg, or an increase in systolic blood pressure of 30 mmHg or more, or in diastolic blood pressure of 15 mmHg or more from the baseline. Pre-eclampsia was defined as high blood pressure plus significant proteinuria (as defined by authors). Ideally, a proteinuria of 1+ or greater by dipstick testing, equal to or greater than 300 mg per 24 hours, or equal to or greater than 500mg per liter is taken as significant proteinuria [15]. Gestational hypertension was defined as high blood pressure developed during pregnancy ± proteinuria (not significant enough to define as pre-eclampsia). Definition of severe pre-eclampsia was also followed as defined by authors usually as systolic blood pressure ≥ 160 and/or diastolic blood pressure ≥110 or more on 2 occasions 4 hours apart plus proteinuria (3+ on urine dipstick) [15]. Eclampsia was defined as the development of convulsions and/or unexplained coma during pregnancy or postpartum in patients with signs and symptoms of preeclampsia after 20 weeks pre-partum and before 48 hours postpartum [15].

### Data abstraction and quality assessment

All the included trials were assessed for methodological quality and outcomes of interest using a standardized form [12]. Data were abstracted for study design, study site, methods of sequence generation, allocation concealment, attrition and primary outcomes of interest. Individual studies were evaluated according to CHERG adaptation of GRADE technique [12,13]. In this method of qualitative evaluation, all RCTs received an initial score of ‘high’ and an observational study as ‘low’. The study scores were adjusted depending on limitations of the study design. Trials with a final grade of ‘high’ or ‘moderate’ and ‘low grade’ were included in the analysis with exclusion of studies with a final grade of ‘very low’[12].

### Quantitative data synthesis

The primary outcomes assessed were maternal mortality, gestational hypertension (± proteinuria), pre-eclampsia, severe pre-eclampsia and eclampsia. Data on neonatal outcomes like neonatal mortality, preterm birth, low birth weight and birth of small-for-gestational age were also extracted. Pooled analyses were conducted where data were available from more than one study for an outcome. The results are presented as risk ratios (RR) and 95% confidence intervals (CIs). The assessment of statistical heterogeneity among trials was done by visual inspection i.e. the overlap of the confidence intervals among the studies, and by the Chi square (P-value) of heterogeneity in the meta-analyses. A low P value (less than 0.10) or a large chi-squared statistic relative to its degree of freedom was considered as providing evidence of heterogeneity. The I^2^ values were also looked into and I^2^ values greater than 50% were taken as substantial and high heterogeneity. In situations of substantial or high heterogeneity being present, causes were explored by sensitivity analysis and random effects model were used. Although random model is not a substitute for a thorough investigation of heterogeneity, it takes an ‘average’ effect from all the included studies compared to fixed models that take the exact contribution from the individual studies [16].It is thus preferred in case of significant heterogeneity in pooled estimate. All analyses were conducted using software Rev Man version 5 [17]. We did a subgroup analysis based on *a priori* hypothesis that calcium supplementation during pregnancy would be more effective in reducing hypertensive disorders in pregnant women who are at increased risk for developing gestational hypertensive disorders. Participants were defined as being at a higher risk of developing hypertension in pregnancy in case of teenage pregnancy, women with previous pre-eclampsia, and women with positive roll over test and/or positive angiotension II sensitivity test [10]. We applied CHERG rules to collective maternal and neonatal mortality and morbidity outcomes related to maternal hypertensive disorders [12]. The purpose of this exercise was to get a point estimate for effectiveness of calcium supplementation during pregnancy in reducing maternal and neonatal mortality due to hypertensive disorders.

## Results

### Trial flow

**Figure 1:**
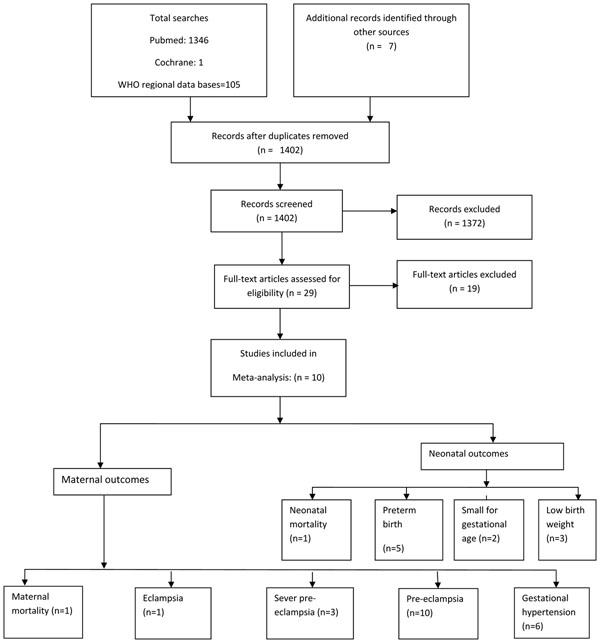
Flow diagram for identification of studies evaluating calcium supplementation during pregnancy for prevention of maternal hypertensive disorders

Literature search of electronic databases, and papers from hand searches yielded a total number of 1402 titles after removal of duplicates (Figure 1). Initially 29 studies were considered for inclusion in the review. Out of these seven studies were excluded due to insufficient data on outcomes of interest [18-24]. Three studies were excluded due to very low grade quality [25-27]. In two trials, calcium was supplemented as therapeutic intervention and not as preventive [28,29]. Two studies were excluded because calcium was supplemented in combination, either with linoleic acid [30] or L-aspartate [31] and it was not possible to separate out their effect from calcium supplementation. Five studies were excluded because they were conducted in developed countries [32-36].Finally 10 studies that met our inclusion criteria were included in the review [37-46].

### Study characteristics

All the included studies were randomized controlled trials with comparison group receiving a placebo in all except in two studies in which participants of comparison group were simply observed as controls [45,46]. Table 1 presents characteristics of included studies. The starting period of calcium supplementation in all the included studies was before 20-32 weeks of gestation and continued till delivery. In three of the included studies [39,40,42], the participants were defined as being at a higher risk of developing hypertension in pregnancy (pregnant teenage girls, women with previous pre-eclampsia or women with positive roll over test). The dose of calcium ranged from 0.5 g/day to 2 g/day. Five of the included studies were from Asia [38,42,43,45,46] and four from South America [37,39-41]. One large multicentre trial was conducted by World Health Organization (WHO) in Argentina, Egypt, India, Peru, South Africa and Vietnam [44].

**Table 1:** Characteristics of included studies

Study (ref)	Country	Target population	Baseline Calcium intake as Low (< 900 mg/day), Adequate (>900g/day), Not specified)	Dose of Supplementation (Cumulative dose)	Duration of supplementation	GRADE quality
Belizan et al 1991[37]	Argentina	Nulliparous pregnant women, < 20 weeks gestation. No comorbid	Low	2 g/day	< 20 weeks of pregnancy till delivery	High
Kumar et al 2009 [38]	India	Primigravida women with gestational age between 12-25 weeks	Low	2 g/day	12-25 weeks of pregnancy till delivery	High
L-Jaramillo et al. 1989[41]	Ecuador	Nulliparous pregnant women, < 24 weeks of gestation. No comorbid	Low	2 g/day	23 weeks of pregnancy till delivery	Moderate
L-Jaramillo et al. 1990[40]	Ecuador	Nulliparous pregnant women in 28-30 weeks of gestation with positive roll over test	Low	2 g/day	28-30 weeks of pregnancy till delivery	Moderate
L-Jaramillo et al.1997[39]	Ecuador	Teenage (< 17.5 years) Nulliparous pregnant women < 20 weeks gestation. No comorbids or addiction	Low	2 g/day	20 weeks of pregnancy till delivery	High
Niromanesh et al. 2001[42]	Turkey	28-32 weeks pregnant women with positive roll over test and with at least one risk factor for pre-eclampsia. No chronic medical condition	Not specified	2 g/day	28-32 weeks of pregnancy till delivery	High
Purwar et al. 1996[43]	India	Nulliparous pregnant women < 20 weeks of gestation. No cormorbid	Low	2 g/day	20 weeks of pregnancy till delivery	High
Villar et al. 2006 [44]	Multicenter trial (Argentina, Egypt, India, Peru, South Africa and Vietnam)	Primiparous women < 20 weeks of gestation. No comorbids	Low	1.5 g/day	From enrollment till delivery	High
Taherian et al. 2002[46]	Iran	Nulliparous pregnant women < 20 weeks of gestation. No comrbids	Low	500 mg/day	From enrollment till delivery	Moderate
Wanchu et al. 2001[45]	India	Nulliparous pregnant women < 20 weeks of gestation. No known comorbids	Low	2 g/day	From enrollment till delivery	Moderate

### Quantitative data synthesis

#### Maternal mortality

Data on cause specific maternal mortality (i.e. due to gestational hypertensive disorders) were not available in any of the included studies. The outcome of all-cause maternal death was reported by only one study [44], with 1 death occurring in the intervention group and 6 deaths in the control group (RR 0.17; 95% CI 0.03-0.76).The overall quality grade for this outcome was that of ‘low’ level due to very low number of events. In the same study, a cumulative outcome for severe maternal morbidity/mortality was also reported. It included all the severe morbidities related to maternal hypertensive disorders that can lead to maternal death [44]. These morbidities include: admission to intensive or special care unit, eclampsia, severe pre-eclampsia, placental abruption, HELLP (hemolysis, elevated liver enzyme and low platelet count) syndrome, renal failure and maternal death. Maternal calcium supplementation during pregnancy showed a significant reduction in the intervention compared to control (RR 0.80; 95 % CI 0.70-0.91). The overall quality grade for this outcome was that of ‘moderate’ level.

#### Eclampsia

One large trial [44] reported outcome on eclampsia. The trials reported 17 cases of eclampsia in 4151 participants of calcium group and 25 cases in 4161 participants of control group giving a relative risk of 0.68 (95 % CI 0.48-0.97). A quality grade of ‘low’ was assigned due to low number (<50 events) of events in the intervention and control group.

#### Severe pre-eclampsia

**Figure 2:**
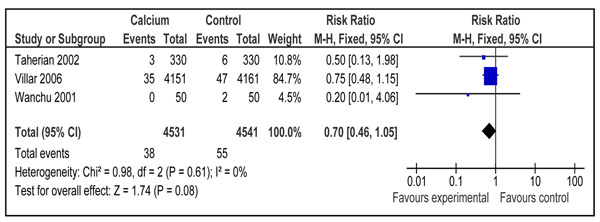
Effect of calcium supplementation during pregnancy on risk of development of severe pre-eclampsia in developing countries:

The outcome of severe pre-eclampsia was reported by three included trials [44-46] with a 30% reduction in calcium group compared to control, however the results were not statistically significant [(RR 0.70; 95% CI 0.46-1.05) (Figure 2). There were a total of 4531 participants in calcium group and 4541 participants in control group. There was no heterogeneity in the pooled data. The overall quality grade for this estimate was that of ‘moderate’ level due to lack of placebo in two studies and confidence interval including unity. We did not perform any subgroup analysis for this outcome due to fewer numbers of studies reporting this outcome.

#### Pre-eclampsia

**Figure 3:**
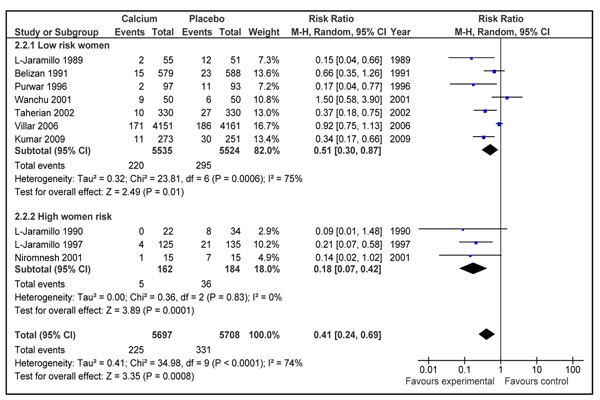
Effect of calcium supplementation during pregnancy on risk of development of pre-eclampsia in developing countries

The impact of calcium supplementation during pregnancy on risk of pre-eclampsia was reported in 10 studies [37-46]. The analysis comprising 5697 women in intervention group and 5708 women in control group showed a reduction of 59% [RR 0.41; 95 % CI 0.24-0.69, random model] in the intervention group compared to control (Figure 3). On visual inspection of the forest plot, five of the included studies were showing a clear benefit. There was a substantial heterogeneity in the pooled data (I^2^=74), so the random models were used. The reduction was more marked in participants with a higher pre-pregnancy risk of developing gestational hypertensive disorders [RR 0.18, 95 % CI 0.07-0.42, random model] compared to that of low risk women [RR 0.51, 95 % CI 0.30-0.87]. The overall quality grade for reduction in risk of pre-eclampsia was that of ‘High’ level.

#### Gestational hypertension (± proteinuria)

**Figure 4:**
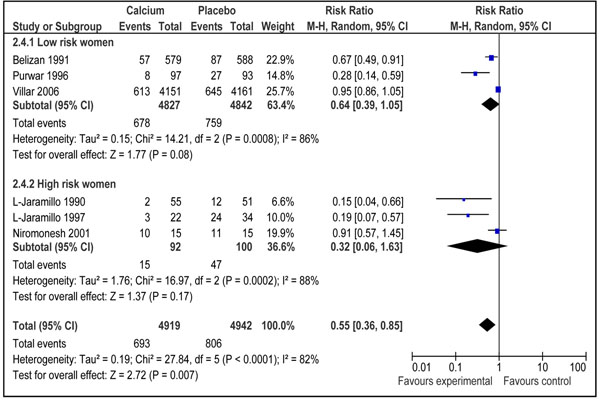
Effect of calcium supplementation during pregnancy on risk of development of gestational hypertension (±proteinuria) in developing countries

The effect of calcium supplementation on gestational hypertension (± proteinuria) was assessed in six studies from developing countries [37,40-44]. A random effect model pooled analysis showed a significant reduction of 45 % in risk of development of gestational hypertension in women receiving calcium supplementation (4919 women in calcium group) as compared to those receiving control (4942 women in control group) [RR 0.55; 95 % CI 0.36-0.85] .On visual inspection of forest plot, four of the included studies were showing a clear benefit in favor of intervention (Figure 4). There was a significant heterogeneity in the pooled data (I^2^=82%) and the random models were used. The overall grade quality for this estimate was that of ‘High’ level. Women who were at higher risk of development of hypertension during pregnancy seems to have a more prominent preventive effect of calcium supplementation [RR 0.32, 95 % CI 0.06-1.63] compared to those at lower risk [RR 0.64, 95 % CI 0.39-1.05], however the results were not statistically significant for both the subgroups (Figure 4).

#### Neonatal outcomes

**Figure 5:**
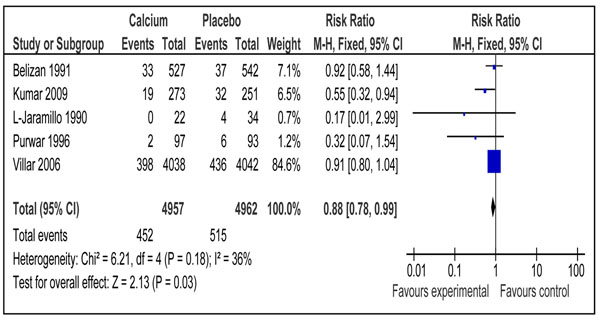
Effect of calcium supplementation during pregnancy on risk of preterm birth in developing countries

**Figure 6:**
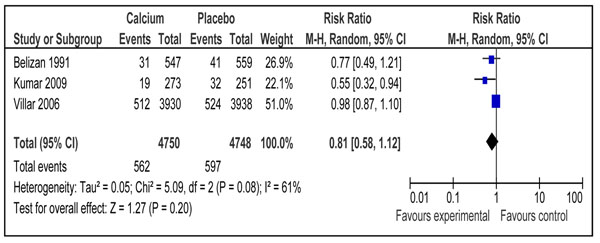
Effect of calcium supplementation during pregnancy on risk of Low Birth Weight (< 2500 g)

One study reported effect of calcium supplementation during pregnancy on neonatal mortality [44]. There was a significant reduction of 30 % in the intervention group compared to placebo (RR 0.70; 95 % CI 0.56-0.88). Data on preterm births were included from five trials [37,38,40,43,44] and the pooled analysis showed a significant reduction of 12% (RR 0.88; 95% CI 0.78-0.99) in the intervention group compared to control (Figure 5). The overall grade for this estimate was that of ‘high level’. The outcome of risk of low birth weight in newborn was reported in three trials [37,38,44] showing no impact of calcium supplementation compared to control (RR 0.81 95% CI 0.58-1.12) (Figure 6). Combined data from two studies [43,44] showed a non-significant reduction in risk of small for gestational babies (RR 0.90; 95 % 0.59-1.38) (data not shown). The overall quality grade for both of the above estimates was that of ‘moderate’ level.

#### Recommendations for LiST

We followed standardized guidelines of Child Health Epidemiology Reference Group (CHERG) to get a point estimate for effectiveness of calcium supplementation during pregnancy in reducing maternal and neonatal mortality related to gestational hypertensive disorders in developing countries [12]. These rules were applied to collective mortality and morbidity outcomes to get a most appropriate estimate for inclusion in the LiST model. The final decision about the best estimate is based on three components 1) the volume and consistency of the evidence 2) the size of risk ratio and 3) the strength of the statistical evidence for an association between the intervention and outcome, as reflected by the p-value [12]. More details about application of CHERG rules are provided in the methods paper [12].

Additional File 1 details the quality assessment of the calcium supplementation trials on maternal and neonatal outcomes. Table 2 presents the application of CHERG rules applied to collective *maternal* mortality and morbidity outcomes. The data on maternal mortality was reported in only one study [44], with a total of 7 events in both arms, which could not be used due to number of events being less than 50 (Rule 1) [12]. Therefore, severe morbidity outcomes were considered. Considering the direction of effect, value of effect size and statistical significance of the estimates, reduction in severe maternal morbidity/mortality was chosen for inclusion in the LiST (Rule 3).This combined outcome was reported by one study and included severe gestational hypertensive related morbidities that can lead to maternal mortality [44]. The qualitative assessment of this estimate was that of ‘moderate’ level however downgraded to ‘low’ to translate it to maternal mortality[47]. To estimate the effectiveness of calcium supplementation during pregnancy on neonatal outcomes, CHERG rules were applied to the outcomes of neonatal mortality, preterm birth, and small for gestational age and low birth weight. One study reported all-cause neonatal mortality which showed significant reduction of 30 % with 37 events in the intervention and 53 events in control group [44]. The overall quality grade for this estimate was that of moderate level which was also downgraded to ‘low’ for translating all-cause into cause specific mortality. This estimate for reduction in all-cause mortality was recommended for inclusion in the LiST as effectiveness of calcium supplementation during pregnancy for neonatal outcomes [12]. Table 3 presents the application of CHERG rules applied to collective neonatal mortality and morbidity outcomes.

**Table 2:** Application of standardized rules to collective mortality and morbidity outcomes to estimate effect of calcium supplementation during pregnancy on *maternal mortality*:

Outcome measure	Studies	Total Events	Reduction (Relative risk)	GRADE quality of pooled estimate	Application of standard rules
Cause specific Maternal Mortality	0	-	-	-	-
All cause maternal mortality	(n=1)	7	83% (RR 0.17; 95% CI 0.02, 1.39)	Low	Rule 1: Do not apply < 50 events
Eclampsia	(n=1)	42	32% reduction (RR 0.68; 95% CI 0.37, 1.26)	Low	Rule 3: Do not apply < 50 events

Severe gestational hypertensive related morbidity/mortality*	**(n=1)**	**376**	**20 %** **(RR 0.80; 95 % CI 0.70-0.91)**	**Moderate** **(→ low)**	**Rule 3 applies** **“*If*** there is low- or very low-quality evidence of effect on cause-specific mortality, ***An*d** there is high- or moderate-quality evidence of effect on serious morbidity… ***Then*** use the smaller of the two effects.” [47]

Severe Pre-eclampsia	(n=3)	93	30% (RR 0.70; 95% CI 0.46, 1.05)	moderate	
Pre-eclampsia	(n=10)	558	59 % (RR=0.41; 95% CI 0.24-0.69)	High	

* Include any one of the following; admission to intensive or special care unit, eclampsia, severe pre-eclampsia, placental abruption, HELLP (hemolysis, elevated liver enzyme and low platelet count) syndrome, renal failure or maternal death.

**Table 3:** Application of standardized rules for choice of final outcome to estimate effect of calcium supplementation during pregnancy on neonatal mortality

Outcome measure	Studies	Total Events	Reduction (Relative risk)	GRADE quality of pooled estimate	Application of standard rules
**All-cause neonatal mortality**	**1**	**90**	**30 %** **(RR 0.56-0.88)**	**Moderate** **(→ low)**	**Rule 1: applies** **“*If*** there is no evidence of effect on cause-specific mortality, ***And*** there is evidence of effect on all-cause mortality… ***Then*** translate all-cause into cause-specific, and downgrade the quality score by one level”[47]

Preterm Birth	5	967	12% (RR 0.88; 95% CI 0.78, 0.99)	High	
Low birth weight (< 2500 g)	3	1159	19 % (RR 0.81; 95 % CI 0.58-1.12]	Moderate	
Small for gestational age	2	84	10 % (RR 0.90; 95 % CI 0.59-1.38)	Moderate	

## Discussion

Role of calcium supplementation during pregnancy in reducing hypertensive disorders has been evaluated before. The review by Hofmeyr et al [8,10] included studies from both developed and developing countries and their pooled estimate had shown that calcium supplementation during pregnancy significantly reduced occurrence of gestational hypertension [RR 0.70, 95% CI 0.57-0.86] and pre-eclampsia [RR 0.48, 95% CI 0.33-0.69]. In the same review, risk of prematurity was also reduced in calcium supplemented group compared to control however the results were not statistically significant [RR 0.81, 95% CI 0.64-1.03]. On the other hand, a review by Trumbo and Ellwood for US Food and Drug Administration (FDA) has shown that the beneficial effects of calcium supplementation during pregnancy cannot be generalized to USA and populations with adequate baseline calcium intake [11]. This conclusion was based on critical evaluation of studies conducted in similar setting as that of USA; however no meta-analysis was performed.

Our results are confirmatory for the above mentioned reviews. If we pool all the studies from both developed and developing countries, the estimates become RR 0.70 (95 % CI 0.57-0.86) for gestational hypertension, RR 0.47 (95 % CI 0.34-0.66) for pre-eclampsia and RR 0.76 (95 % CI 0.59-0.97) for risk of preterm birth. Estimates for gestational hypertension and pre-eclampsia are similar to that of Hofmyer et al. [10] however the results for risk of preterm birth became statistically significant. This is due to addition of new study from India by Kumar et al which had shown a significant effect in reduction in risk of preterm birth [38]. When we separately pooled the results of studies from developed countries only [32-36], the estimate came to be RR 0.77 (95% CI 0.57, 1.03, random model) for gestational hypertension, RR 0.52 (95 % CI 0.27, 1.00, random model) for pre-eclampsia and RR 0.63 (95 % CI 0.33, 1.19, random model) for preterm birth (data not shown).This shows that calcium supplementation did not have any significant effect on risk of gestational hypertensive disorders in developed countries as is shown in the descriptive review of FDA [11].

What could be the explanation of protective effect of calcium supplementation during pregnancy in developing countries and no effect in developed countries? The first and the foremost is the difference in baseline calcium intake. In most of the included studies from developing countries the baseline intake was low (Table 1),while it was adequate in most of the studies from developed countries as reported in review by FDA [11]. Low calcium intake has been hypothesized to cause increase in blood pressure by stimulating the release of parathyroid hormone and/or renin which leads to increased intracellular calcium concentration in vascular smooth muscle cells and causes vasoconstriction [48]. Role of calcium supplementation in reducing hypertensive disorders in pregnancy can possibly be explained by reduction in parathyroid calcium release and intracellular calcium concentration, thereby reducing smooth muscle contractility and promoting vasodilatation [49]. Calcium supplementation could also prevent preterm labor and delivery by reducing uterine smooth muscle contractility [36] directly and indirectly by increasing magnesium levels [22]. The second explanation could be prevalent malnutrition in developing countries. It had been proposed that hormones involved in blood pressure control are altered during malnutrition and can lead to significant morbidity in malnourished pregnant women [47,50].

**Figure 7:**
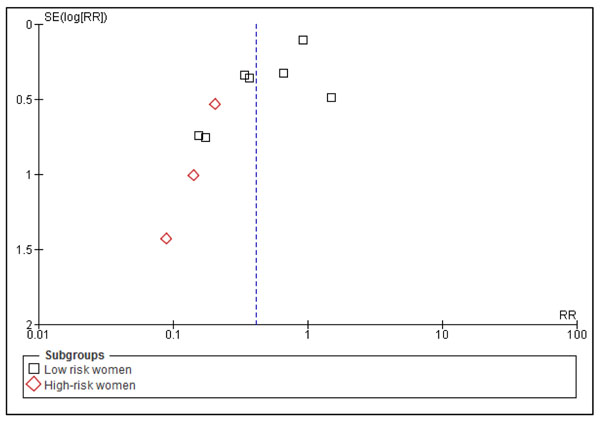
Funnel plot of studies evaluating effect of calcium supplementation during pregnancy in reducing risk of pre-eclampsia in developing countries.

Our review has shown that calcium supplementation during pregnancy reduces all gestational hypertensive related disorders. The reduction in pre-eclampsia was more than 50% in the present review. There was a great deal of heterogeneity in the pooled estimate which can primarily be explained by difference in the effect size, with significant results more strongly present in smaller trials (Figure 3). The two major contributors to heterogeneity were studies by Villar et al. 2006 [44], the largest of included studies and study by Wanchu et al. 2001 [45] the second smallest study in the pooled data. Another explanation for this heterogeneity was the increased effect of intervention in trials with high risk women included, and on sub-group analysis the most marked reduction in pre-eclampsia was in the above group, and no heterogeneity was reported in the results (Figure 3). The funnel plot for risk of pre-eclampsia was not very symmetric (Figure 7). Studies with low SE contributed the most to the pooled effect size however two studies with high SE had the highest protective effect. Publication bias may be one of the reasons of this asymmetry of the funnel plot however this may also be due to smaller sample size of some of the included studies.

Maternal mortality is a relatively rare event [2]. It is very difficult to design studies powered enough to detect a significant effect of an intervention on maternal mortality. According to CHERG rules, we had recommended reduction in severe maternal morbidity/mortality as a proxy for effectiveness of calcium supplementation during pregnancy in reducing maternal mortality related to gestational hypertensive disorders, for inclusion in the LiST model. This composite outcome included gestational hypertensive related severe maternal morbidities that can lead to maternal death [44]. This approach of reporting potentially fatal gestational hypertensive related maternal morbidities have also been adopted in antioxidant trials for prevention of pre-eclampsia [51]. The overall grade evidence for this outcome was that of ‘moderate’ level which was downgraded to ‘low’ for its recommendation as a proxy for maternal mortality. We also applied CHERG rules to neonatal outcomes. Considering mortality and morbidity outcomes, effect size for all-cause neonatal mortality was chosen for inclusion in the LiST based on its statistical significance and quality grade. The overall quality grade was that of ‘moderate’ level which was downgraded to ‘low’ for translating all-cause onto cause specific mortality[47]. The beneficial effect of calcium supplementation in reducing neonatal mortality seems to be related to reduction in preterm birth and birth asphyxia. There was a significant reduction of 12 % (95 % CI 1% to 22%) in preterm birth in calcium supplemented group compared to control (Figure 5). This pathway can be related to severe gestational hypertensive morbidities (e.g. eclampsia) which can lead to preterm birth and birth asphyxia and calcium supplementation has shown reduction in all severe morbidities (Additional File 1). It is important to note that maternal calcium supplementation during pregnancy is not only effective in reducing neonatal mortality but also morbidities later in childhood. A review by Bergel and Barros had reported that offspring of women who were supplemented with calcium during pregnancy had low incidence of hypertension in childhood [52].

Our review has certain limitations. In two of the included studies [45,46], the comparison group did not receive the placebo but the participants were simply observed as controls. This could have biased the results in favor of intervention[16]. We did not look at the side effects related to calcium supplementation during pregnancy. The previous reviews, however, have shown that it is not associated with any particular harmful effects [10].

Findings of this review and those of previous reviews gave conclusive evidence on effectiveness of calcium supplementation during pregnancy in reducing maternal gestational hypertensive related disorders in populations with low baseline calcium intake [8,11]. Future research should focus on delivery platforms, regimens and programmatic aspect of the intervention. It would be relevant to assess the bioavailability of calcium when delivered for example via dietary modification or food fortification. It is important for example to determine effectiveness of calcium supplementation by dietary modification at places where baseline calcium intake is from dairy products compared with those where it is mostly taken in from vegetarian sources. It is also important to calculate an internationally accepted value to define adequacy given the large variations in calcium recommendations in different countries of the world.

Implementation of recommendation of calcium supplementation to all pregnant women in developing countries poses a major challenge to policy-makers and program managers of these countries. It is important to take steps for procurement of the preparation, storage, distribution, quality-control, and compliance assurance with daily supplements to large numbers of pregnant women. It is also important to consider cultural, financial, and educational barriers to changing policy and lessons should be learnt from practices of previous programs like iron+folic acid supplementation schemes in these countries. Lack of infrastructure and poor compliance were considered as few of the major barriers in implementation of these programs in these countries [53]. Issues of cost effectiveness should also be considered and weighed for increasing the calcium intake by dietary modification or food fortification. Increasing dietary calcium intake may seem to be an easier intervention than calcium supplementation, although availability of dairy products in many countries may not be sufficient to fulfill the need. Alternatively, targeted food fortification with calcium may be a feasible intervention, especially for high-risk women, who may not be targeted for individual calcium supplementation because they do not come for antenatal care services [54].

## Conclusion

Calcium supplementation during pregnancy leads to a reduction of 59 % (95 % CI 31 % to 76 %) in risk of pre-eclampsia, 45 % (95 % CI 15 % to 64 %) in risk of development of gestational hypertension and 12 % (95 % CI 1% to 22 %) in risk of preterm birth in developing countries. Calcium should be supplemented to all women during pregnancy in developing countries.

## Competing interests

We do not have any financial or non-financial competing interests for this review.

## Authors' contributions

Professor Zulfiqar A Bhutta conceived the idea and secured support for the review.  Dr Aamer Imdad and Dr Afshan Jabeen undertook the literature search, data extraction and wrote the manuscript under the supervision of Professor Bhutta.

## Supplementary Material

Additional File 1 Quality assessment of calcium supplementation during pregnancy on maternal and neonatal outcomes Click here for file
